# Organized screening programmes for breast and cervical cancer in 17 EU countries: trajectories of attendance rates

**DOI:** 10.1186/s12889-018-6155-5

**Published:** 2018-11-06

**Authors:** Maria Michela Gianino, Jacopo Lenzi, Marco Bonaudo, Maria Pia Fantini, Roberta Siliquini, Walter Ricciardi, Gianfranco Damiani

**Affiliations:** 1 Department of Public Health Sciences and Pediatrics, Università di Torino, Via Santena 5 bis, 10126 Turin, Italy; 2 Department of Biomedical and Neuromotor Sciences, Alma Mater Studiorum - Università di Bologna, Via Ugo Foscolo 7, 40123 Bologna, Italy; 3 Fondazione Policlinico Universitario ‘Agostino Gemelli’ IRCSS, Largo Agostino Gemelli 8, 00168 Roma, Italy; 4 Istituto di Sanità Pubblica, Università Cattolica del Sacro Cuore, Largo Francesco Vito 1, 00168 Roma, Italy

**Keywords:** Breast cancer, Cervical cancer, Healthcare, Organized screening, Socioeconomic inequalities, Socioeconomic variables, Trend

## Abstract

**Background:**

The aim was to analyse participation trajectories in organised breast and cervical cancer screening programmes and the association between socioeconomic variables and participation.

**Methods:**

A pooled, cross-sectional, time series analysis was used to evaluate secondary data from 17 European countries in 2004–2014.

**Results:**

The results show that the mammographic screening trend decreases after an initial increase (coefficient for the linear term = 0.40; *p* = 0.210; 95% CI = − 0.25, 1.06; coefficient for the quadratic term = − 0.07; *p* = 0.027; 95% CI = − 0.14, − 0.01), while the cervical screening trend is essentially stable (coefficient for the linear term = 0.39, *p* = 0.312, 95% CI = − 0.42, 1.20; coefficient for the quadratic term = 0.02, *p* = 0.689, 95% CI = − 0.07, 0.10). There is a significant difference among the country-specific slopes for breast and cervical cancer screening (SD = 16.7, *p* < 0.001; SD = 14.4, *p* < 0.001, respectively). No association is found between participation rate and educational level, income, type of employment, unemployment and preventive expenditure. However, participation in cervical cancer screening is significantly associated with a higher proportion of younger women (≤ 49 years) and a higher Gini index (that is, higher income inequality).

**Conclusions:**

In conclusion three messages: organized cancer screening programmes may reduce the socioeconomic inequalities in younger people’s use of preventive services over time; socioeconomic variables are not related to participation rates; these rates do not reach a level of stability in several countries. Therefore, without effective recruitment strategies and tailored organizations, screening participation may not achieve additional gains.

## Background

Screening for breast and cervical cancer is strongly related with a reduction in cancer mortality [1, 2].

Screening strategies differ between countries. Some countries have organized screening that systematically tests all women in the defined target group, either on a national or regional level [3].

Opportunistic screenings, in which the women’s participation is a result of a recommendation made by a healthcare practitioner or of their own choice, are adopted by other countries [4].

An assessment of these screening programmes shows that the coverage of the target population and positive response to screening are higher in population-based programmes than in opportunistic screening [5]. The assessment of these screening programmes also shows that organized screening programmes for breast and cervical cancer based on an active recruitment strategy are better than opportunistic screenings as far as increasing participation rates are concerned [6, 7].

Because of their increased population coverage, follow-up and quality control, population-based programmes effectively reduce mortality and control the inappropriate use of screening tests [8–11], whereas opportunistic screening is strongly criticized for using community resources without any demonstrable effect on cancer rates [12].

Some studies highlight the fact that social and economic factors correlate with use of cancer screening, and socioeconomic inequity in cancer screening is dramatically reduced in countries with organized screening programmes compared with countries without them [5, 13, 14].

Thus, implementing organised screening programmes has been recommended by the European Community [15], and many member states have done so. To date, most European countries have developed population-based screening programmes for both breast and cervical cancers, but they differ in terms of organisational characteristics, implementation stage, and coverage [16–18].

To our knowledge, there is no study that focuses on this type of data from several EU countries to analyse participation rates in organised breast and cervical cancer screening programmes.

The aims of this study are as follows: i) to analyse participation rates in organised breast and cervical cancer screening programmes in 17 European countries; ii) to describe the annual variations in screening attendance rate during 2004–2014 and to determine the trend over time; and iii) to systematically analyse the association between socioeconomic variables and participation rates.

## Methods

We conducted a pooled, cross-sectional, time series analysis of 17 European countries over the period 2004–2014. The countries included in the study were: Belgium, Czech Republic, Denmark, Estonia, Finland, France, Germany, Iceland, Ireland, Italy, Luxembourg, Netherlands, Norway, Slovakia, Slovenia, Sweden, and United Kingdom. These countries and years were chosen based on data availability.

We obtained official, secondary data from the Organization for Economic Co-operation and Development (OECD), Eurostat and Global Economic Monitor. The indicators considered in the present study are listed in Table 1, which includes the definition and source of each item. All indicators were selected for females except the economic indicators (i.e. indicators: 3; 4; 5a; 5b), for which sex stratification was not available.

**Table 1:** Indicators, definitions and data sources

#	Indicator	Definition	Source
1a.	Breast cancer screening, programme data	Females aged 50–69 screened (%)	OECD Health Statistics 2017
1b.	Cervical cancer screening, programme data	Females aged 20–69 screened (%)	OECD Health Statistics 2017
2a; 2b; 2c; 2d; 2e; 2f; 2 g; 2 h; 2i; 2 l	Demographic structure	Persons, Female, ten age classes (25–29; 30–34; 35–39, 40–44; 45–49;50–54;55–59;60–64;65–69 years) over 20–69 (%)	OECD Health Statistics 2017
2 m; 2n; 2o; 2p	Demographic structure	Persons, Female, four age classes (50–54;55–59;60–64;65–69 years), over 50–69 (%)	OECD Health Statistics 2017
3.	Income of households	Euro per inhabitant, disposable income. Household income includes every form of income (e.g., salaries and wages, retirement income, near cash government transfers like food stamps, and investment gains) available for spending and saving after income taxes.	EUROSTAT 2017
4.	Gini - index of income equality	The Gini coefficient is a measure of income distribution and is used to determine income inequality in a population. It ranges from 0 to 100%, with 0% representing perfect equality (i.e., every resident has the same income), and 100% representing perfect inequality (i.e., one resident earns all the income). The index of income equality refers to disposable income, post taxes and transfers, in a working age population aged 18–65.	OECD Health Statistics 2017
5a.	Preventive Care All Financing Schemes	Per capita, constant prices, constant PPPs, OECD base year – US Dollar 2010. Financing Schemes identify the main types of financing arrangements through which health services are paid for and obtained by people and include the following: i) Voluntary health insurance under which the access to health services is at the discretion of private actors through paid premiums; ii) Out-of-pocket payments under which the access to health services is at the discretion of private actors by a direct payment for services from the household primary income or savings; iii) Government healthcare financing schemes that involve financing arrangements to ensure access to all citizens/residents, or for a specific group of the population (e.g., the poor) defined by law/government regulation through domestic revenues of government (primarily taxes); iv) Compulsory health insurance that involves a financing arrangement to ensure access to healthcare for specific population groups through mandatory participation and eligibility based on the payment of health insurance contributions by or on behalf of the individuals concerned.	OECD Health Statistics 2017
5b.	Preventive Care Government schemes and compulsory contributory health care financing schemes	Per capita, constant prices, constant PPPs, OECD base year - US Dollar 2010	OECD Health Statistics 2017
6a; 6b; 6c; 6d; 6e	Educational attainment level	Female, five age classes (20–24; 25–34; 35–44, 45–54; 55–64 years), Less than primary, primary and lower secondary education (levels 0–2) (%)	EUROSTAT 2017
6f; 6 g; 6 h; 6i; 6 l	Educational attainment level	Female, five age classes (20–24; 25–34; 35–44, 45–54; 55–64 years), Upper secondary and post-secondary non-tertiary education (levels 3 and 4) (%)	EUROSTAT 2017
6 m; 6n; 6o; 6p; 6q	Educational attainment level	Female, five age classes (20–24; 25–34; 35–44, 45–54; 55–64 years), Tertiary education (levels 5–8) (%)	EUROSTAT 2017
7	Unemployment	Female (% of female labour force) (modelled International Labour Organization-ILO estimate)	GLOBAL ECONOMIC MONITOR 2017
8	Self-employed	Female (% of female employment)	GLOBAL ECONOMIC MONITOR 2017

Abbreviations: OECD, Organization for Economic Co-operation and Development

### Statistical analyses

Statistical methods used in this study were drawn from previously published works [19, 20].

First, a time trend analysis was performed by using a fixed-effects polynomial regression on the annual participation rates in breast and cervical cancer screening programmes. Second, a pooled time-series cross-section analysis was performed to assess the association between the screening participation rates (indicators 1a and 1b, i.e., dependent variables) and a set of independent variables (indicators 2a to 8) over the 11-year study period. We chose to fit a linear model because the dependent variables were confirmed to be normally distributed by performing the Shapiro-Wilk and Shapiro-Francia tests [21].

To avoid model overfitting, indicators 2c to 2 l were halved by collapsing age groups, while secondary and tertiary education levels (6f to 6q) were merged because they yielded similar results. Additionally, no results were reported for indicators 2a and 2b, 2 m, and 6a to 6e because they make 100 when added to the indicators 2c to 2 l, 2n to 2p, and 6f to 6q, respectively. Due to potential over-fitting and multi-collinearity, we separately examined the relationship between all the remaining dependent and independent variables, resulting in 21 distinct fixed-effects models. The proportion of missing observations per model ranged from 0% (educational attainment) to approximately 25% (unemployment/self-employment), and listwise deletion was used because all data appeared to be missing at random (data not shown).

All data were analysed using the Stata software package, version 13 (StataCorp. 2013, Stata Statistical Software: Release 13; StataCorp LP, College Station, TX, USA). For all analyses, the significance level was set at *p* < 0.05.

## Results

### Screening attendance rates

For breast screening, all countries have had national or regional screening programmes since 2004, except for Denmark and Slovenia, whose programmes began as pilot programmes in 2009 and 2008, respectively.

**Fig. 1:**
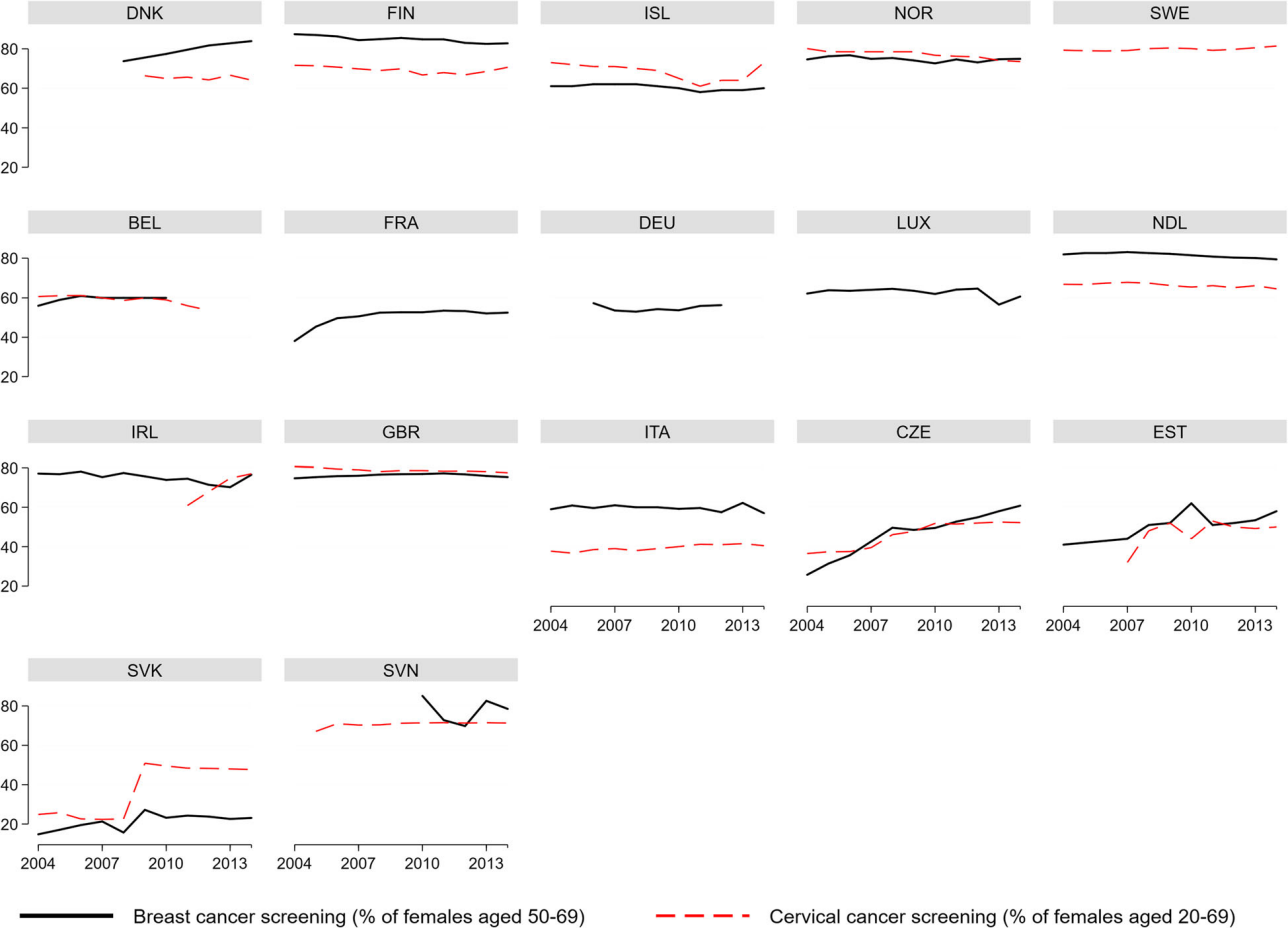
Participation rates (%) in breast cancer and uterine cervix screening in 17 EU countries from 2004-2014^A. A^ Breast screening data not available for Sweden. Uterine cervix screening data not available for Ireland between 2007 and 2011. Abbreviations: BEL, Belgium; CZE, Czech Republic; DNK, Denmark; EST, Estonia; FIN, Finland; FRA, France; DEU, Germany; ISL, Iceland; IRL, Ireland; ITA, Italy; LUX, Luxembourg; NDL, Netherlands; NOR, Norway; SVK, Slovakia; SVN, Slovenia; SWE, Sweden; GBR, United Kingdom

There is great variability between the countries: in Slovakia, participation in mammographic screening does not reach 30%, while in Finland the percentage is always higher than 80%. Countries such as Belgium, the Czech Republic, Estonia, France, Germany, Iceland, Italy and Luxembourg do not reach the 70% participation threshold outlined in the European Guidelines (Fig. 1).

Three countries have no cervical screening programmes (France, Germany and Luxembourg). Other countries have screening programmes since 2004, except for Estonia (since 2008) and Ireland with a nationwide programme since 2007. There is high variability, and no programme reaches the 85% threshold defined in the European guidelines. The largest attendance rates are registered in Sweden, the UK and Norway (Fig. 1).

### Annual trends in screening attendance rates

**Fig. 2:**
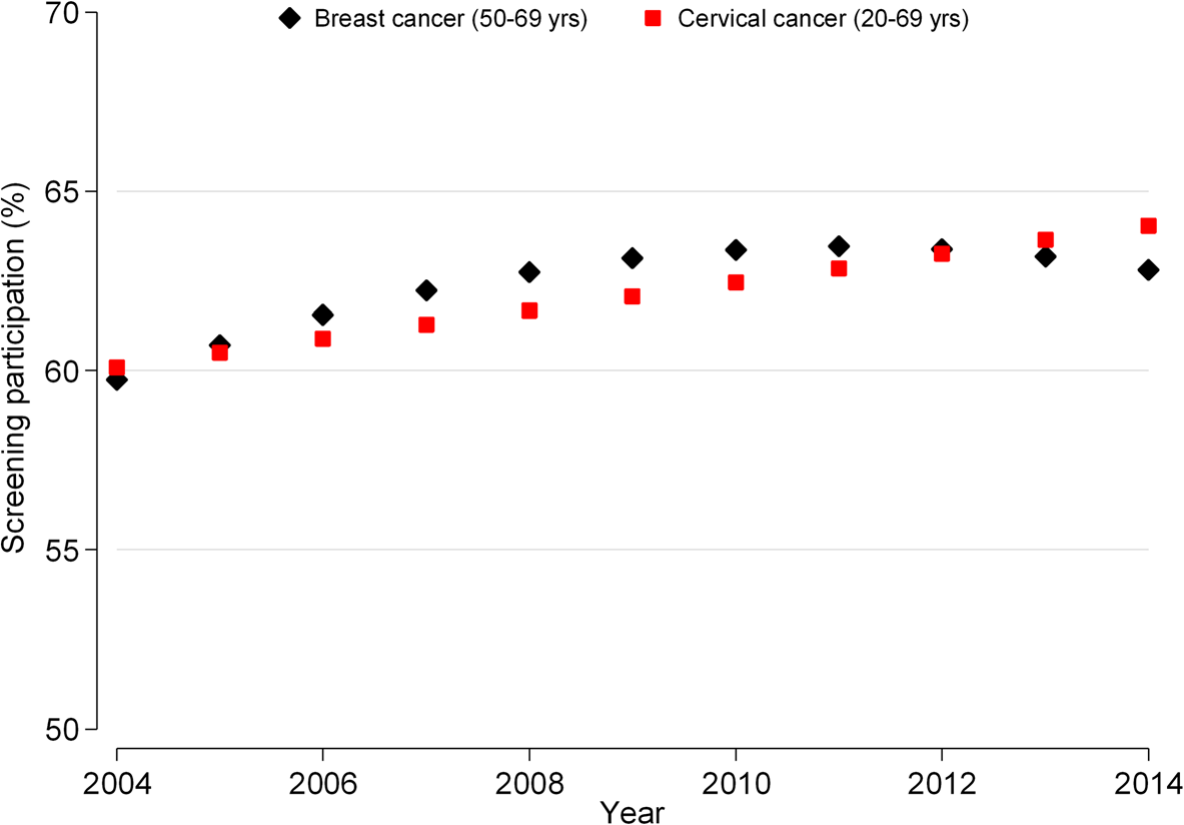
Estimates from the fixed-effects regression analysis. Overall participation rate (%) in breast cancer and cervical cancer screening in 17 EU countries from 2004 to 2014

The linear trend of participation in mammographic screening is not significant (coefficient for the linear term = 0.40; *p* = 0.210; 95% CI = − 0.25, 1.06) but is significantly “curved” (coefficient for the quadratic term = − 0.07; *p* = 0.027; 95% CI = − 0.14, − 0.01). When the coefficient for the linear term is near zero and the coefficient for a quadratic term is negative and significant, it means that the time trend is concave (apex at the top) and that the values at the beginning and the end of the study period are similar. As confirmed in Fig. 2, which illustrates the expected participation rates derived from the fixed-effects polynomial model, there is a slight decrease in participation in breast cancer screening after an initial slight increase.

Table 2 shows the average annual rates of change in percentages of women subjected to breast cancer screening in European countries during the analysed period. The trend of the individual countries is discordant and it seems to be unconnected to the geographic area: 5 nations (Czech Republic, Denmark, Estonia, France and Slovakia) experienced a significant increase in screening participation, while 3 countries (Finland, Iceland and Netherland) had a significant decline in participation rates; all this countries belong to different geographic areas. The regression model shows that there is a significant difference among the country-specific slopes for breast cancer screening (standard deviation [SD] = 16.7, *p* < 0.001).

**Table 2:** Participation rates (%) for breast cancer and uterine cervix screening in 17 European countries, 2004–2014

Country	Breast cancer screening (pop. Aged 50–69)				Cervix cancer screening (pop. Aged 20–69)			
	First available	Last available	Average	*P*	First available	Last available	Average	*P*
	% (Year)	% (Year)	ann. Change (Slope)		% (Year)	% (Year)	ann. Change (Slope)	
Northern Europe								
Denmark	73.7	83.9	1.70	0.006	66.3	64.1	−0.44	0.431
	(2008)	(2014)			(2009)	(2014)		
Finland	87.4	82.8	−0.46	< 0.001	71.6	70.6	−0.10	0.084
	(2004)	(2014)			(2004)	(2014)		
Iceland	61.0	60.0	−0.10	0.010	73.0	73.0	0.00	0.142
	(2004)	(2014)			(2004)	(2014)		
Norway	74.6	74.9	0.03	0.132	80.1	73.5	−0.66	< 0.001
	(2004)	(2014)			(2004)	(2014)		
Sweden	n/a	n/a	n/a	n/a	79.3	81.4	0.21	0.008
					(2004)	(2014)		
Central Europe								
Belgium	56.0	60.0	0.67	0.177	60.7	53.7	−0.88	0.006
	(2004)	(2010)			(2004)	(2012)		
France	38.1	52.5	1.44	0.029	n/a	n/a	n/a	n/a
	(2004)	(2014)						
Germany	57.3	56.3	−0.17	0.845	n/a	n/a	n/a	n/a
	(2006)	(2012)						
Luxembourg	62.2	60.7	−0.15	0.218	n/a	n/a	n/a	n/a
	(2004)	(2014)						
Netherlands	81.9	79.4	−0.25	0.002	66.9	64.6	−0.23	0.006
	(2004)	(2014)			(2004)	(2014)		
British Isles								
Ireland	77.1	76.5	−0.06	0.072	60.9	77.0	5.37	0.019
	(2004)	(2014)			(2011)	(2014)		
United Kingdom	74.7	75.3	0.06	0.294	80.6	77.5	−0.31	< 0.001
	(2004)	(2014)			(2004)	(2014)		
Southern Europe								
Italy	59.0	57.0	−0.20	0.486	37.7	40.5	0.28	< 0.001
	(2004)	(2014)			(2004)	(2014)		
Eastern Europe								
Czech Republic	25.7	60.8	3.51	< 0.001	36.5	52.2	1.57	< 0.001
	(2004)	(2014)			(2004)	(2014)		
Estonia	41.0	58.0	1.70	< 0.001	32.0	50.0	2.57	0.186
	(2004)	(2014)			(2007)	(2014)		
Slovakia	14.8	23.1	0.83	0.002	24.9	47.7	2.28	< 0.001
	(2004)	(2014)			(2004)	(2014)		
Slovenia	85.1	78.5	−1.65	0.876	67.1	71.3	0.47	0.095
	(2010)	(2014)			(2005)	(2014)		

For uterine cervix screening programmes, neither the linear nor the quadratic terms are significant (coefficient for the linear term = 0.39, *p* = 0.312, 95% CI = − 0.42, 1.20; coefficient for the quadratic term = 0.02, *p* = 0.689, 95% CI = − 0.07, 0.10). Participation is essentially stable until the end of the decade (Fig. 2).

Table 2 shows the average annual rates of change in percentages of women subjected to cervical cancer screening in European countries during the analysed period. The trend of the individual countries is again discordant and again seems to be unconnected to the geographic area: 5 nations (Czech Republic, Ireland, Italy, Slovakia and Sweden) experienced a significant increase in screening participation, while 4 countries (Belgium, Netherland, Norway and UK) had a significant decline in participation rates. The regression model shows that there is a significant difference among the country-specific slopes for cervical cancer screening as well (SD = 14.4, *p* < 0.001).

### Socioeconomic variables and screening attendance rates

Table 3 shows the results of the regression analysis, which evaluates the impact of demographic, social and economic factors of the population on screening attendance rates. The only significant results relate to the demographic structure of the female population and the distribution of income (GINI index). Specifically, there is a 5.18% increase in screening participation when the percentage of women aged 30–39 is 1% higher. In addition, screening participation increases by 3.46% when the percentage of women aged 40–49 over those aged 20–69 years is 1% higher.

**Table 3:** Results of the regression analysis

Regressor	Breast cancer	Cervical cancer
	screening	Screening
	Per 100	Per 100
	inhabitants	Inhabitants
% Persons, Female, aged 55–59 over 50–69	2.29	–
	(1.07)	
% Persons, Female, aged 60–64 over 50–69	1.79	–
	(0.93)	
% Persons, Female, aged 65–69 over 50–69	1.42	–
	(1.01)	
Time effect	0.87	
	(0.566)	
*R* ^*2*^	0.954	
Countries	16	
Average obs.per country	8.9	
% Persons, Female, aged 30–39 over 20–69	–	5.18*
		(1.11)
% Persons, Female, aged 40–49 over 20–69	–	3.46*
		(1.16)
% Persons, Female, aged 50–59 over 20–69	–	3.29
		(2.05)
% Persons, Female, aged 60–69 over 20–69	–	1.9
		(1.93)
Time effect		1.77
		(0.081)
*R* ^*2*^		0.942
Countries		13
Average obs. per country		8.2
Mean income of households (€ in thousands)	1.36	0.61
	(0.83)	(1.01)
Time effect	0.68	1.1
	(0.741)	(0.372)
*R* ^*2*^	0.95	0.901
Countries	14	13
Average obs. per country	8.4	8.4
Gini index (%)	0.35	1.52*
	(0.22)	(0.35)
Time effect	1.52	1.36
	(0.145)	(0.216)
*R* ^*2*^	0.943	0.89
Countries	16	14
Average obs. per country	7.5	7.6
Preventive Care All Financing Scheme ($)	0.0	−0.05
	(0.03)	(0.04)
Time effect	1.7	1.41
	(0.089)	(0.185)
*R* ^*2*^	0.938	0.891
Countries	16	14
Average obs. per country	8.6	8.9
Preventive Care Government and Compulsory insurance schemes ($)	−0.07	− 0.15
	(0.05)	(0.09)
Time effect	2.37	2.3
	(0.014)	(0.018)
*R* ^*2*^	0.939	0.899
Countries	16	14
Average obs. per country	8.6	8.9
Educational attainment level % Female, 20–24 years,	–	0.06
Education levels 3–8		0.15
Time effect		1.65
		(0.103)
*R* ^*2*^		0.902
Countries		14
Average obs. per country		9.1
Educational attainment level % Female, 25–34 years,	–	−0.31
Education levels 3–8		(0.45)
Time effect		1.46
		(0.165)
*R* ^*2*^		0.896
Countries		14
Average obs. per country		9.7
Educational attainment level % Female, 35–44 years,	–	−0.36
Education levels 3–8		(0.29)
Time effect		1.76
		0.076
*R* ^*2*^		0.898
Countries		14
Average obs. per country		9.7
Educational attainment level % Female, 45–54 years,	−0.19	0.11
Education levels 3–8	(0.27)	0.24
Time effect	1.72	0.61
	(0.083)	(0.800)
*R* ^*2*^	0.942	0.894
Countries	16	14
Average obs. per country	9.7	9.7
Educational attainment level % Female, 55–64 years,	−0.56	−0.09
Education levels 3–8	(0.32)	0.26
Time effect	4.17	0.82
	(< 0.001)	(0.610)
*R* ^*2*^	0.949	0.894
Countries	16	14
Average obs. per country	9.7	9.7
Unemployment, % of female labour force	0.09	0.3
	(0.39)	(0.34)
Time effect	0.53	0.99
	(0.814)	(0.446)
*R* ^*2*^	0.863	0.896
Countries	16	14
Average obs. per country	7.2	7.4
Self-employed, % of female employment	0.75	−0.13
	(0.94)	(0.98)
Time effect	0.48	1.35
	(0.850)	0.24
*R* ^*2*^	0.963	0.896
Countries	16	14
Average obs. per country	7.2	7.4

*Notes:* Robust standard errors are given in parentheses under the coefficients, and *p*-values are given in parentheses under the *F*-statistics of time effect. The individual coefficient with an asterisk (*) is significant at the 5% level

Additionally, when the percentage of the Gini coefficient increases, a 1.52% increase in cervical screening attendance is observed.

## Discussion

The aim of this study was to analyse participation rates in organised breast and cervical cancer screening programmes in 17 European countries, to describe the annual variations in screening attendance rate during 2004–2014, to determine the trend over time and to systematically analyse the association between socioeconomic variables and participation rates.

Our results show that the countries that had already in 2004 crossed the 70% threshold of participation to breast cancer screening, as prescribed by the guidelines, continued to experience membership rates above the threshold even in 2014, while the countries that showed rates lower than 70% in 2004 or the first year that data screening were available did not reach the recommended threshold in 2014. No countries reached the 85% attendance rate in cervical cancer screening.

Inferential statistical analysis provides information on the trend by showing the annual change in screening attendance rates. All 17 European countries experienced a “curved” trend in breast cancer screening and an essentially stable trend for uterine cervix screening during the 11-year study period. These results demonstrate that, despite two Cochrane reviews that found interventions encouraging breast and cervical cancer screening were effective in increasing participation, these programmes do not seem sufficient [6, 7].

The analysed countries contribute to these trends with different average annual change rates that are unconnected to the geographic area.

Several reasons may justify the different slopes of the countries. The literature highlights that the reasons behind the failure or success of cancer screening participation are two-fold. First, there are determinants on the supply side [22]. Second, there are obstacles on the demand side because certain sections of the target population decline the use of the screening offered [22]. On the supply side, the specialized literature highlights that the key components of successful programmes include the following: a high level of target population coverage, identification of strategies of attendance, personal invitations for all eligible persons [23, 24] and the availability of trained personnel and adequate equipment [25, 26]. These components are the basis, for example, of the significant growth in cervical screening in Ireland, where a national screening programme called “Cervical Check” was introduced. This programme extends screening to 100% of the target population, uses an organized call and recall system of invitation, has a dedicated smeartaker training unit that runs training courses for new providers and includes regular updates for established smeartakers, and increases the availablity of quality assured services such as laboratory services and colposcopy for the screening [27]. These components are also the basis of the constant high level of breast cancer screening participation rates in British Isles. Indeed, all English and Irish women aged between 50 and 70, who are registered with a General Practitioner, are invited personally to attend for screening mammography by their local breast screening unit. Each woman receives a timed appointment, and if a woman does not attend that appointment she is sent a second timed appointment.

Similarly, these components play a role in the significant reduction in cervical screening participation in Belgium, where formal cervical cancer screening exists in only one of the three geographically separated regions (Flemish), and the invitations are not yet sent to all of the target population [28, 29].

These components also seem to justify, on the one hand, the significant growth of participation in breast screening in some Eastern European countries, such as in the Czech Republic and Slovakia, and on the other hand, the achievement of modest coverage that is below the standard ‘acceptable level’ (70%). In the Czech Republic, a personal recruitment system through gynaecologists and general practitioners was introduced for breast screening and was reinforced by media campaigns, but it has no established centralized system of direct invitation. In Slovakia, the screening programme became strictly invitational in 2004, and it targeted age groups for screening, being limited at first to 45–59 year olds and then representing women aged 50–65 years starting in 2007. However, a widely information to women and an improvement the availability of a quality service it still needs to be implemented for raising the participation rate (national cancer strategy) [30].

Obstacles on the demand side may justify the slopes of other countries. In particular, France has increased the participation to a breast screening programme; however, by the end of the years under consideration in this study, it did not reach the threshold of 70% as set by the European Union [31]. A suggestion is provided by Ferrat et al., which states there are several barriers that affect attendance to screening programmes, and one of these is the perceptions of women with regard to the benefits of prevention [32]. Italy also noticed an increase in participation to cervical screening thanks to the initiatives of the National Health Service [33], even though it did not reach the threshold defined by the European Union. In fact, according to the study by Damiani et al., the perception of the low efficacy of cancer screening, the anxiety about the results and the fear of cancer continue to affect participation in screening [34].

According to our results, some socioeconomic variables, such as level of education, income and type of employment and unemployment, are not related to the rate of participation in screening.

Educational level did not seem to affect participation, but women with higher levels of education had slightly lower attendance compared with women with the lowest level of education. One possible reason for this trend may be that more educated women are more likely to use private services. Other studies have found no association between education and participation [13, 35–37], somewhat lower participation by women with both the lowest and highest levels of education [38], or an inverse correlation between level of education and screening attendance [39].

Employment status may be a good measure of socioeconomic status. Among socioeconomic groups based on employment status, women who are not currently employed or who are self-employed are less likely to attend a screening. The latter is in keeping with other studies [40–43].

Mean household income is not related to the rate of screening participation. This may be because most European health systems offer free or inexpensive testing, reducing financial barriers to screening; this is in keeping with previous studies demonstrating that cost has no effect on participation [44].

No association is found between preventive expenditure and screening participation. This suggests that health systems must focus and improve the organization of screenings, given the evidence that screening reduces breast and cervical cancer-related mortality. More importantly, it suggests that health systems need to choose interventions that are more effective at increasing participation rates. The literature shows that some types of interventions are more effective, specifically among underserved communities, and highlights that other types of interventions do not have sufficient evidence of effectiveness [45].

The lack of association between the above-cited socioeconomic variables and the rate of screening participation suggests that screening programme success depends on other variables such as recruitment strategies and tailored organization. This hypothesis is in keeping with findings from previous studies that show evidence of the impact of different strategies on enhancing attendance within a given programme [7, 26, 46]. A review showed that the most effective recruitment strategies are those that act on behavioural change by developing tailored messages that may break down the barriers holding back screening participation and consequently support women’s individual decisions [47]. Additionally, population-based programmes should involve all health actors who are in contact with the target population, especially the primary care physicians. Because primary care physicians are usually the first medical practitioner contacted by patients and have more familiarity with them, they may play a key role in promoting screening [26]. By acting on these other variables, it may be that the trend of breast cancer screening, which showed a slight decrease in participation after an initial slight increase, could be affected.

An important finding in this study is that participation in cervical screening increased when the relative size of young populations (30–49 years) increased during the 11-year study period. This is not surprising and is consistent with previous studies demonstrating that advanced age is associated with decreased screening [14, 48].

Another important and more unexpected result is the positive impact that the GINI index had on participation in cervical screening. Our findings show that screening attendance is greater in societies with younger population age structure (i.e. % of women aged 30–49 higher) and greater income inequality (i.e. GINI index higher). This result, based on a time series analysis during the 11-year period, suggests that organized screening programmes may be effective in reducing socioeconomic inequalities in screening over time. This suggestion is supported by a review that concludes that a longer period of time is needed to see the positive effects of an organized screening programme [26]. Indeed, according to the review, there is no evidence to date that an organized programme may be effective in reducing socio-economic inequalities in screening attendance [26] but this evidence arises from studies that are biased because they assess effectiveness after only a short period following implementation [26]. It has been postulated in a previous study [49] that public health interventions initially increase inequalities for coverage and reduce inequalities later; this because people with a high socio-economic position are more likely to be involved [49], and the poor only gain greater access to the interventions later [49].

The main weakness of this study is that the analysis was conducted at the national level, and the state-wide distribution was not analysed. An analysis carried out within the national context may highlight particular groups of women who fail to attend screenings and, importantly, increase understanding of the factors that influence a woman’s decision to participate in cervical screenings.

We analysed the trend using a variety of indicators of socioeconomic status. This approach is supported by the specialized literature [50, 51], and we consider it a strength of our study that we used different indicators.

Some preliminary conclusions can be drawn from this study. First, it appears that organized cancer screening programmes may over time reduce the socioeconomic inequalities found in the utilization of preventive services by younger people. Second, socioeconomic variables are not related to participation rates and, third, all countries do not reach the European standard level set at 85% for cervical screening, and most countries do not reach the 70% level that is acceptable by the standards for breast screening. Moreover, there is evidence that the participation rates did not reach a level of stability in several countries during the 11-year study period.

This suggests that without effective recruitment strategies, which also reduce barriers, and without an organization focused on the characteristics of the specific country, screening participation may not achieve additional gains.
